# Supplementary material for: A survey of the kinome pharmacopeia reveals multiple scaffolds and targets for the development of novel anthelmintics
Source: Sci Rep. 2021 Apr 28;11:9161. doi: 10.1038/s41598-021-88150-6 (PMC8080662; doi:10.1038/s41598-021-88150-6)
Supplement: Supplementary file 6 — Supplementary Legends. [file 41598_2021_88150_MOESM6_ESM.pdf]

Supplementary Figure 1. Structural comparison of EGFR/LET-23 homology model and LET-23 crystal structure. (A) Homology model of only the *C. elegans* LET-23, which is modeled after an EGFR crystal structure complexed with AMP-PNP (PDB: 2ITX, AMP-PNP in yellow). The *C. elegans* LET-23 residues that diverge from Human EGFR that also have side chains oriented towards the active site are highlighted in dark pink. Residues are labeled according to position in human EGFR, with the first letter indicating the identity of the human residue and the latter indicating the identity of the *C. elegans* residue. (B) The crystal structure of only the LET-23 kinase domain complexed with AMP-PNP (PDB: 5WNO, AMP-PNP in yellow) with the same residues highlighted in (A) here are highlighted in light pink. The orientation of these key residues towards the active site is consistent between the LET-23 homology model and crystal structure. The homology model and crystal structure of LET-23 are overlaid in (C).

Supplementary Figure 2. Structures and alignments for druggable nematode essential kinase targets. In all alignments, residues that are proximal to the inhibitor binding site with side-chains facing inwards are highlighted on the alignment. Grey residues were examined but do not differ between *C. elegans* and humans. Divergent residues are highlighted in dark pink *(C. elegans* residue) and light pink (human residue). Yellow highlights those residues from different species that differ in identity from both the *C. elegans* kinase and the human ortholog of interest in these key positions. Some alignments include additional human paralogs where we also color residues yellow that differ from *C. elegans* and the relevant human ortholog. Residues of interest that differ between nematodes and vertebrates are labeled with their position in the human kinase on the alignment and shown in dark and light pink on the structure above. Those residues of interest indicated with a star on the alignment are located within 5Å of the bound inhibitor in any of the *C. elegans* models examined.

(A) Structure and alignment of EGFR and LET-23. Structure of PDB:1XKK (Human EGFR) in green with Lapatinib bound (in yellow), aligned to *C. elegans* LET-23 homology model in blue. The human and *C. elegans* kinase domains share 44% identity. All residues that are proximal to the inhibitor binding site with side-chains facing

inwards are highlighted on the alignment (identified from PDB structures 1XKK, 2ITX, 3W2S, 4G5J and associated *C. elegans* homology models). The orthologous LET-23 sequence from the parasitic nematode *B. malayi* was modeled to the human EGFR structure (PDB: 1XKK) to confirm the favourable position of the identified divergent residues within the inhibitor binding site. Residues of interest are indicated on the structures in dark pink (nematode residue) and light pink (human residue).

(B) Structure and alignment of MEK1 and MEK-2. Structure of PDB: 3PP1 (Human MEK1) in green with allosteric inhibitor TAK-733 bound (in orange) aligned to *C. elegans* MEK-2 homology model in blue above. Structure of PBD:5EYM (Human MEK1) with ATP-competitive inhibitor BI-847325 bound (in yellow) aligned to *C. elegans* MEK-2 homology model below. The purple arrow in the BI-847325-bound structure alignment highlights the *C. elegans* leucine residue that may sterically hinder BI-847325 from binding *C. elegans* MEK-2. This steric hindrance is further visualized in the surface representations of the Human MEK1 crystal structure and *C. elegans* MEK-2 homology model. The human and *C. elegans* kinase domains share 60% identity. All residues proximal to the ATP-competitive inhibitor binding site (identified in PDB structures 5EYM, 5HZE) and allosteric site (identified from PDB structures 3EQH, 3PP1) with side-chains facing inwards are highlighted on the alignment. The orthologous MEK-2 sequence from the parasitic nematode *B. malayi* was modeled to the human MEK-1 structures (PDB: 3PP1 above, PDB: 5EYM below) to confirm the favourable position of the identified divergent residues within the inhibitor binding site. Residues of interest are indicated on the structures in dark pink (nematode residue) and light pink (human residue).

(C) Structure and alignment of PLK1 and PLK-1. Structure of PDB: 2RKU (Human PLK1) in green with BI2536 bound (in yellow), aligned to *C. elegans* PLK-1 homology model in blue. The human and *C. elegans* kinase domains share 64% identity. All residues that are proximal to the inhibitor binding site with side-chains facing inwards are highlighted on the alignment (identified from PDB structures 2RKU, 3FC2, 2YAC, 4J52 and associated *C. elegans* homology models). The orthologous PLK-1 sequence

from the parasitic nematode *B. malayi* was modeled to the human PLK1 structure (PDB:2RKU) to confirm the favourable position of the identified divergent residues within the inhibitor binding site. Residues of interest are indicated on the structures in dark pink (nematode residue) and light pink (human residue).

(D) Structure and alignment of BRAF and LIN-45. Structure of PDB: 5CSW (Human BRAF) in green with dabrafenib bound (in yellow), aligned to *C. elegans* LIN-45 homology model in blue. The human and *C. elegans* kinase domains share 62% identity. All residues that are proximal to the inhibitor binding site with side-chains facing inwards are highlighted on the alignment (identified from PDB structures 5CSW, 5CT7 and associated *C. elegans* homology models). Residues of interest are indicated on the structure above in dark pink (*C. elegans* residue) and light pink (human residue).

(E) Structure and alignment of AURKB and AIR-2. Structure of PDB: 5EYK (*Xenopus laevis* AURKB) in green with BI-847325 bound (in yellow), aligned to *C. elegans* AIR-2 homology model in blue. The human and *C. elegans* kinase domains share 64% identity. All residues that are proximal to the inhibitor binding site with side-chains facing inwards are highlighted on the alignment (identified from human structure PDB: 4AF3 and *X. laevis* structure PDB: 5EYK and associated *C. elegans* homology models). Residues of interest are indicated on the structure above in dark pink (*C. elegans* residue) and light pink (*X. laevis* residue).

(F) Structure and alignment of MTOR and LET-363. Structure of PDB: 4JSX (Human MTOR) in green with Torin 2 bound (in yellow), aligned to *C. elegans* LET-363 homology model in blue. The human and *C. elegans* kinase domains share 62% identity. All residues that are proximal to the inhibitor binding site with side-chains facing inwards are highlighted on the alignment (identified from PDB structures 4JSX, 4JSV and associated *C. elegans* homology models). Residues of interest are indicated on the structure above in dark pink (*C. elegans* residue) and light pink (human residue).

(G) Structure and alignment of CDK1 and CDK-1 Structure of PDB: 4Y72 (Human CDK1) in green with inhibitor bound (in yellow), aligned to *C. elegans* CDK-1 homology model in blue. The human and *C. elegans* kinase domains share 67% identity. All residues that are proximal to the inhibitor binding site with side-chains facing inwards are highlighted on the alignment (identified from PDB structures 4Y72, 6GU4 and associated *C. elegans* homology models). Residues of interest are indicated on the structure above in dark pink (*C. elegans* residue) and light pink (human residue).

(H) Structure and alignment of MAPK14 and PMK-1. Structure of PDB: 6SFO (Human MAPK14) in green with SR-318 bound (in yellow), aligned to *C. elegans* PMK-1 homology model in blue. The human and *C. elegans* kinase domains share 71% identity. All residues that are proximal to the inhibitor binding site with side-chains facing inwards are highlighted on the alignment (identified from PDB structures 6SFO, 3ZS5, 1DI9, 1KV2 and associated *C. elegans* homology models). Residues of interest are indicated on the structure above in dark pink (*C. elegans* residue) and light pink (human residue).

(I) Structure and alignment of SRC and SRC-1. Structure of PDB: 6ATE (Human SRC) in green with inhibitor bound (in yellow), aligned to *C. elegans* SRC-1 homology model in blue. The human and *C. elegans* kinase domains share 63% identity. All residues that are proximal to the inhibitor binding site with side-chains facing inwards are highlighted on the alignment (identified from PDB structures 6ATE, 4MXO and associated *C. elegans* homology models). Residues of interest are indicated on the structure above in dark pink (*C. elegans* residue) and light pink (human residue).

(J) Structure and alignment of IGF1R and DAF-2. Structure of PDB: 5FXS (Human IGF1R) in green with inhibitor bound (in yellow), aligned to *C. elegans* DAF-2 homology model in blue. The human and *C. elegans* kinase domains share 47% identity. All residues that are proximal to the inhibitor binding site with side-chains facing inwards are highlighted on the alignment (identified from PDB structures 5FXS, 4D2R and

associated *C. elegans* homology models). Residues of interest are indicated on the structure above in dark pink (*C. elegans* residue) and light pink (human residue).

(K) Structure and alignment of STK10 and GCK-4. Structure of PDB: 6EIM (Human STK10) in green with GW683134A bound (in yellow), aligned to *C. elegans* GCK-4 homology model in blue. The human and *C. elegans* kinase domains share 56% identity. All residues that are proximal to the inhibitor binding site with side-chains facing inwards are highlighted on the alignment (identified from PDB structures 6EIM, 4EQU and associated *C. elegans* homology models). Residues of interest are indicated on the structure above in dark pink (*C. elegans* residue) and light pink (human residue).

(L) Structure and alignment of CSNK1D and KIN-20. Structure of PDB: 5MQV (Human CSNK1D) in green with inhibitor bound (in yellow), aligned to *C. elegans* KIN-20 homology model in blue. The human and *C. elegans* kinase domains share 80% identity. All residues that are proximal to the inhibitor binding site with side-chains facing inwards are highlighted on the alignment (identified from PDB structures 5MQV, 5OKT and associated *C. elegans* homology models). Residues of interest are indicated on the structure above in dark pink (*C. elegans* residue) and light pink (human residue).

(M) Structure and alignment of GSK3B and GSK-3. Structure of PDB: 6HK3 (Human GSK3B) in green with inhibitor bound (in yellow), aligned to *C. elegans* GSK-3 homology model in blue. The human and *C. elegans* kinase domains share 82% identity. All residues that are proximal to the inhibitor binding site with side-chains facing inwards are highlighted on the alignment (identified from PDB structures 6HK3, 5HLN and associated *C. elegans* homology models). Residues of interest are indicated on the structure above in dark pink (*C. elegans* residue) and light pink (human residue).

(N) Structure and alignment of BUB1 and BUB-1. Structure of PDB: 6F7B (Human BUB1) in green with BAY-1816032 bound (in yellow), aligned to *C. elegans* BUB-1 homology model in blue. The human and *C. elegans* kinase domains share 30% identity. All residues that are proximal to the inhibitor binding site with side-chains facing

inwards are highlighted on the alignment (identified from PDB structures 6F7B, 4QPM and associated *C. elegans* homology models). Residues of interest are indicated on the structure above in dark pink (*C. elegans* residue) and light pink (human residue).

(O) Structure and alignment of CSNK2A1 and KIN-3. Structure of PDB: 3R0T (Human CSNK2A1) in green with inhibitor bound (in yellow), aligned to *C. elegans* KIN-3 homology model in blue. The human and *C. elegans* kinase domains share 86% identity. All residues that are proximal to the inhibitor binding site with side-chains facing inwards are highlighted on the alignment (identified from PDB structures 3R0T, 3NSZ and associated *C. elegans* homology models). Residues of interest are indicated on the structure above in dark pink (*C. elegans* residue) and light pink (human residue).

(P) Structure and alignment of CSNK1A1 and KIN-19. Structure of PDB: 6GZD (Human CSNK1A1) in green with inhibitor bound (in yellow), aligned to *C. elegans* KIN-19 homology model in blue. The human and *C. elegans* kinase domains share 90% identity. All residues that are proximal to the inhibitor binding site with side-chains facing inwards are highlighted on the alignment (identified from PDB structure 6GZD and associated *C. elegans* homology model). Residues of interest are indicated on the structure above in dark pink (*C. elegans* residue) and light pink (human residue).

Supplementary Figure 3. 4AQ SAR highlighting favorable structural features for nematode activity *in vivo.* Screen phenotypes induced by inhibitor exposure including lethality (Let), embryonic lethality (Emb), larval arrest (Lva) and sterility (Ste) are shown. The resulting population growth defects are indicated by the colour coded scale (nb, no bacteria remaining in the well).

Supplementary Figure 4. Conservation of essential nematode-specific kinases across species. Matrices show the percent sequence identity between the kinase domain of the *C. elegans* essential nematode-specific kinase and that of the most similar kinase found across nematode species along with the kinase domain sequence of the best human kinase match and its ortholog across vertebrate species. *C. elegans*

kinases from nematode-specific families (A-P) and nematode-expanded families (Q-Z) are shown. Sequence identity matrices for the kinase domain of the well-conserved kinases MEK1/MEK-2, PLK1/PLK-1 and EGFR/LET-23 are included for comparison (AA-AC). Kinase sequences were identified using NCBI BLASTP. Percent identity matrices were generated using Clustal Omega.

Supplementary Table 1. Kinases Targeted by Inhibitor Screen Hits

Supplementary Table 2. Essential Nematode-Specific Kinases

Supplementary File S1. *C. elegans* Vertebrate Kinase Inhibitor Screen Results.
S1A: Summary of 191 hits from kinase inhibitor screen
S1B: Full screen results for LOPAC library kinase inhibitors
S1C: Full screen results for OICR kinase inhibitor library
S1D: Full screen results for APExBIO Discovery Probe kinase inhibitor library
S1E: Full screen results for PKIS1 and PKIS2

Supplementary File S2. EGFR and PLK1 inhibitors included in screening set.
S2A: Commercial EGFR inhibitor screening results
S2B: Screening results for PKIS inhibitor scaffolds targeting EGFR
S2C: Commercial PLK1 inhibitor screening results
S2D: Screening results for PKIS inhibitor scaffolds targeting PLK1
